# White Matter Hyperintensity in Elderly Patients with Diabetes Mellitus Is Associated with Cognitive Impairment, Functional Disability, and a High Glycoalbumin/Glycohemoglobin Ratio

**DOI:** 10.3389/fnagi.2017.00220

**Published:** 2017-07-06

**Authors:** Yoshiaki Tamura, Yoshiyuki Kimbara, Takuya Yamaoka, Ken Sato, Yuki Tsuboi, Remi Kodera, Yuko Chiba, Seijiro Mori, Yoshinori Fujiwara, Aya M. Tokumaru, Hideki Ito, Takashi Sakurai, Atsushi Araki

**Affiliations:** ^1^Departments of Diabetes, Metabolism, and Endocrinology, Tokyo Metropolitan Geriatric HospitalTokyo, Japan; ^2^Research Team for Social Participation and Community Health, Tokyo Metropolitan Institute of GerontologyTokyo, Japan; ^3^Department of Diagnostic Radiology, Tokyo Metropolitan Geriatric HospitalTokyo, Japan; ^4^Center for Comprehensive Care and Research on Memory Disorders, National Center for Geriatrics and GerontologyAichi, Japan

**Keywords:** cognitive impairment, diabetes mellitus, glucose variability, instrumental activities of daily living, white matter hyperintensities

## Abstract

**Aims:** Although evidence has accumulated that white matter hyperintensity (WMH) is associated with the deterioration of cognitive function and impairment of activities of daily living (ADL), the clinical relevance of WMH in elderly patients with diabetes mellitus (DM) is not still clear. The aim of this study was to examine whether WMH volume is associated with ADL and cognitive function and whether glucose control and glucose variability can affect WMH volume in these patients.

**Methods:** This cross-sectional study investigated the associations of WMH with cognitive function and instrumental ADL (IADL), as well as metabolic and vascular risk factors in a total of 178 elderly patients with diabetes. The study assessed WMH volumes and the functional status of cognition and IADL. WMH volumes were evaluated by obtaining axial T2-weighted and fluid-attenuated inversion recovery sequence images on brain magnetic resonance imaging and assessing the images using Software for Neuro-Image Processing in Experimental Research.

**Results:** We found a significant association between WMH volumes and Mini-Mental State Examination (MMSE) scores (*p* = 0.039) and between WMH and IADL status (*p* = 0.006). Furthermore, we found significant relations of large WMH volumes with a high glycoalbumin/glycohemoglobin ratio (GA/HbA1c) (*p* < 0.001). Large WMH volumes were also found to be associated with a low body mass index (*p* = 0.014) and a low diastolic blood pressure (*p* = 0.024), but not with HbA1c. Multiple regression analysis showed that high GA/HbA1c, which reflects high glucose variability, was a significant determining factor for large WMH volumes. We also found that GA/HbA1c was negatively associated with both MMSE (*p* = 0.036) and IADL (*p* < 0.001).

**Conclusion:** GA/HbA1c, which is a marker of glucose variability, was independently associated with WMH volumes, which could lead to the decline of cognition and IADL in elderly patients with DM.

## Introduction

White matter hyperintensity (WMH) is a type of lesion in white matter that is detected with magnetic resonance imaging (MRI), showing high intensity in both T2-weighted and fluid-attenuated inversion recovery (FLAIR) sequence images (Tamura and Araki, 2015). The pattern in MRI images is thought to reflect microvessel damage (Matsusue et al., 2006). It is well known that the greatest risk factor for WMH is hypertension and aging (Dufouil et al., 2001; Honda et al., 2015). It is controversial whether diabetes mellitus (DM) is related to WMH volumes. Some prospective studies have shown that DM is associated with the progression of WMH volumes (Taylor et al., 2003; Gouw et al., 2008), whereas the results of some studies did not support a significant association (den Heijer et al., 2003; Schmidt et al., 2004).

Recently, WMH has been demonstrated to be associated with cognitive dysfunction and impairment of activities of daily living (ADL) (Debette and Markus, 2010; Kreisel et al., 2013), which are the major components of geriatric syndrome. However, as few studies have targeted elderly patients with DM, the importance of WMH on the progression of geriatric syndrome in these patients is unknown. In addition, the risk factors that accelerate WMH volume increase in elderly patients with DM have not yet been clarified.

We hypothesized that WMH is associated with cognitive impairment and ADL dysfunction in elderly patients with DM similar to that in the general population. In the assessment of factors affecting WMH volumes in DM patients, we especially focused on glucose variability. Glucose variability is known to induce oxidative stress and endothelial dysfunction (Torimoto et al., 2013), and it has been shown that postprandial glucose excursions are well correlated with the risk of cardiovascular mortality, regardless of the fasting glucose levels (Nakagami and DECODA Study Group, 2004). Thus, we calculated the glycoalbumin (GA) to glycohemoglobin (HbA1c) ratio (GA/HbA1c) by dividing GA by HbA1c because GA/HbA1c can sensitively represent glucose variability (Ogawa et al., 2012). We also hypothesized that GA/HbA1c is associated with WMH volume in elderly patients with DM.

To test our two hypotheses, we performed a cross-sectional study in 178 elderly patients with DM and investigated the relations between WMH and cognitive function, instrumental ADL (IADL), GA/HbA1c, and other clinical factors.

## Materials and Methods

### Subjects

Elderly (≥65 years of age) patients with DM who presented between October 2013 and June 2016 were included in this study. DM was diagnosed based on the following criteria: highest value of recorded HbA1c ≥ 6.5% or a history of taking antidiabetic medications. Patients with any history of symptomatic stroke or severe diseases were excluded. This study was carried out in accordance with the recommendations of the ethics committee of Tokyo Metropolitan Geriatric Hospital with written informed consent from all subjects. All subjects gave written informed consent in accordance with the Declaration of Helsinki.

### HbA1c and GA Measurements

Blood samples were extracted ad lib. HbA1c (National Glycohemoglobin Standardization Program [NGSP]) was measured using high-performance liquid chromatography, and GA was measured using an enzymatic method.

### Brain MRI

Magnetic resonance imaging images were obtained using 1.5-T MR scanners and included T2-weighted and axial FLAIR sequence images. The images were obtained using GE Signa HDxt (Milwaukee, WI, United States). The slice settings were as follows: 20–22 slices with 5-mm thickness and an interslice gap of 2 mm were obtained in parallel with the anterior commissure–posterior commissure line, covering a maximum of 154 mm and ranging from the vertex down to the lower end of the pons. Eight-channel head coils were used for MR signal acquisition and transmission. The parameters of the T2-weighted sequence were as follows: repetition time (TR), 4300 ms; effective echo time (TEef), 90 ms; echo train length (ETL), 16; field of view (FOV), 220 mm; acquisition matrix, 256 × 288; number of acquisitions (NA), 2. The parameters of the FLAIR sequence were as follows: TR, 10,000 ms; TEef, 100 ms; inversion time (TI), 2500 ms; ETL, 16; FOV, 220 mm; acquisition matrix, 192 × 288; NA, 1.

### Evaluation of WMH Volumes

White matter hyperintensity volumes were evaluated using Software for Neuro-Image Processing in Experimental Research (SNIPER), as previously described (Admiraal-Behloul et al., 2005). WMH volumes were calculated as the total volume in the cerebrum and were divided in two parts (periventricular hyperintensity [PVH] and deep WHM [DWMH]), which were distinguished using SNIPER. Considering the individual variations in skull size, each WMH volume was normalized by dividing it by the patient’s respective intracranial volume (IC) and was described as WMH/IC, PVH/IC, or DWMH/IC (%).

### Evaluation of Cognitive Function and ADL

Cognitive function was tested using the Mini-Mental State Examination (MMSE). IADL was evaluated using the IADL subscaleof the Tokyo Metropolitan Institute of Gerontology Index of Competence, as shown previously (Koyano et al., 1991). This is a questionnaire comprising 13 questions, including three subscales for IADL, intellectual activity, and social roles. The scores of the IADL subscale range from 0 to 5 points.

### Statistical Analysis

Mini-Mental State Examination scores were divided into the following two categories: normal (27–30 points) and cognitive impairment (≤26). IADL scores were divided into the following three categories: 5, 4, and 0–3 points. Common logarithms of WMH/IC and PVH/IC were compared between MMSE and IADL categories using the unpaired *t*-test and analysis of variance, with Tukey’s test as a *post hoc* analysis, respectively. As GA/HbA1c was not normally distributed, we used the Mann–Whitney and Kruskal–Wallis analyses to evaluate GA/HbA1c between MMSE and IADL categories, respectively. Correlations of two continuous variables were analyzed using Spearman’s rank correlation. In multiple regression analysis to identify the determining factor for WMH, we defined WMH/IC as the outcome value, and age, sex, body mass index (BMI), GA/HbA1c, and diastolic pressure as the independent values. Further, to identify the determining factor for MMSE and IADL decline, binomial logistic regression analyses were performed. In these analyses, MMSE and IADL categories were defined as outcome values. IADL scores were divided in two categories (4–5 and 0–3 points), and age, sex, and common logarithms of WMH/IC and GA/HbA1c were substituted as independent values. All statistical analyses were performed using the SPSS Statistics 20 software package (IBM Corp., Armonk, NY, United States). In all comparisons, the significance level was set at *p* < 0.05.

## Results

### Baseline Profiles of the Patients

The baseline profiles of the patients are shown in **Table 1**. The median age was 77 years, and the median HbA1c and disease duration were 7.5% and 15 years, respectively. The median BMI and systolic and diastolic blood pressures were 23.8 kg/m^2^ and 131 mmHg and 70 mmHg, respectively. The median GA value was 21.5%, and the median GA/HbA1c was 2.85. The rates of patients taking antidiabetic and antihypertensive medications were 92.1 and 64.6%, respectively. In our study population, over 130 patients participated in an interview and in MMSE and ADL testing.

**Table 1:** Clinical characteristics of the study subjects.

Age (y)	77 (73–81)
Male (%)	41.6
Disease duration (years) (*n* = 163)	15 (8.0–24.0)
BMI (kg/m^2^) (*n* = 144)	23.8 (21.2–26.6)
Systolic blood pressure (mmHg) (*n* = 177)	131 (120–142)
Diastolic blood pressure (mmHg) (*n* = 176)	70 (61–78)
HbA1c (%)	7.5 (6.8–8.6)
GA (%) (*n* = 176)	21.5 (18.7–26.5)
GA/HbA1c (*n* = 176)	2.85 (2.56–3.20)
TC (mg/dL) (*n* = 177)	171 (149–194)
TG (mg/dL)	113 (79–148)
HDL-cholesterol (mg/dL)	50 (41–60)
eGFR (mL/min/1.73 m^2^) (*n* = 151)	57 (48–68)
Antidiabetic drugs (%)	92.1
Antihypertensive drugs (%)	64.6
Statin (%)	56.7
MMSE (*n* = 134)	28.5 (26.8–30.0)
IADL (*n* = 136)	5.0 (5.0–5.0)
MR imaging	
IC (mL)	1374 (1282–1482)
WMH (mL), % of IC	6.4 (2.8–13.0) 0.46
PVH (mL), % of IC	5.6 (2.6–11.5) 0.41
DWMH (mL), % of IC	0.4 (0.1–1.0) 0.03
PAR (mL), % of IC	996 (926–1080) 72.4
CSF (mL), % of IC	374 (335–416) 27.6
VCL (mL), % of IC	51 (42–66), 3.8

*Data for continuous variables are presented as medians (25–75th percentiles). GA, glycoalbumin; TC, total cholesterol; TG, triglyceride; eGFR, estimated glomerular filtration rate; MMSE, Mini–Mental State Examination; IADL, instrumental activities of daily living; MR, magnetic resonance; IC, intracranial; PVH, periventricular hyperintensity; DWMH, deep WMH; PAR, parenchyma; CSF, cerebrospinal fluid; VCL, ventricular.*

### Association of WMH with Cognitive Function and IADL

**FIGURE 1:**
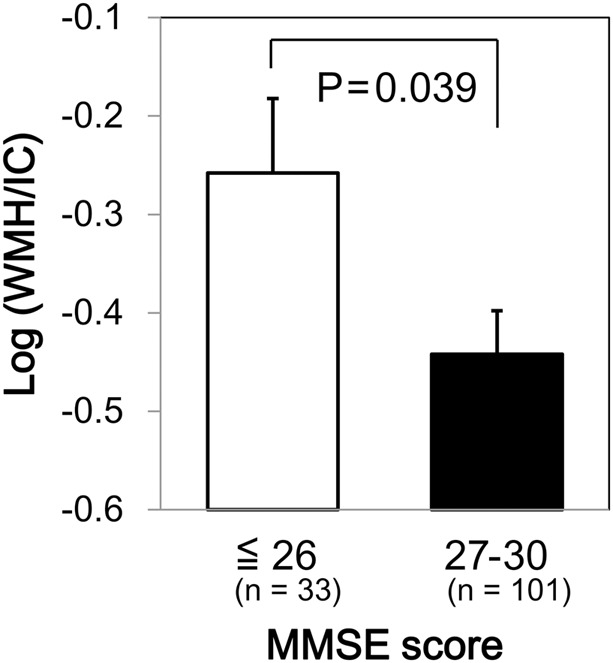
Comparison of common logarithms of the percentage of total white matter hyperintensity (WMH) divided by intracranial volume (WMH/IC) among Mini–Mental State Examination (MMSE) score categories. Bars and error bars represent mean and standard error, respectively.

**FIGURE 2:**
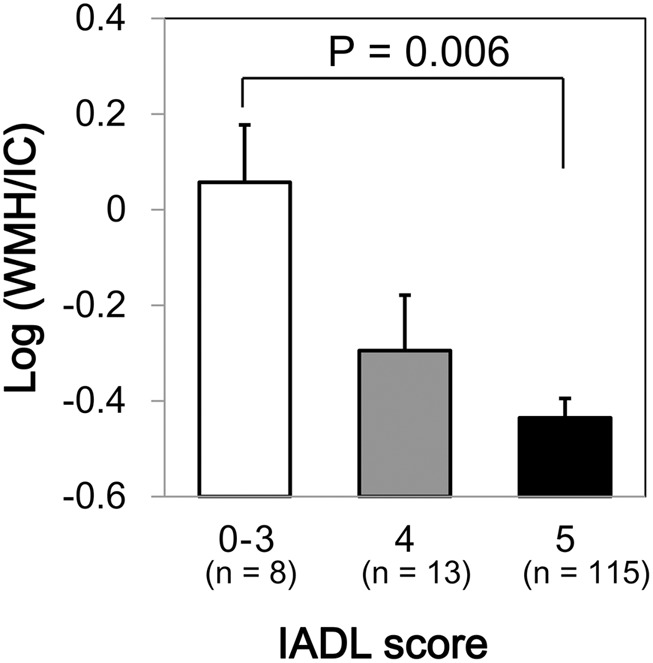
Comparison of common logarithms of the percentage of total WMH divided by intracranial volume (WMH/IC) among instrumental activities of daily living (IADL) score categories. Bars and error bars represent mean and standard error, respectively.

We found a significant difference in WMH volumes between the MMSE categories (*p* = 0.039 for total WMH/IC and *p* = 0.029 for PVH/IC; **Figure 1**). However, there were no significant correlations between DWMH/IC and MMSE (data not shown). Similarly, we found significant negative correlations of WMH/IC and PVH/IC with the IADL categories (*p* = 0.006 for total WMH/IC and *p* = 0.004 for PVH/IC; **Figure 2**). However, DWMH/IC was not related to IADL (data not shown).

### Association between WMH and GA/HbA1c

**FIGURE 3:**
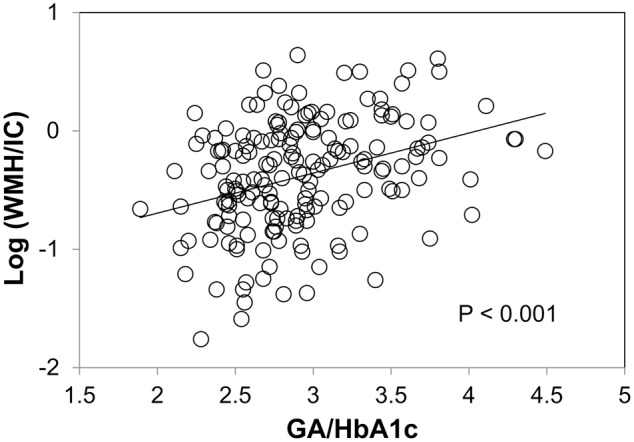
Correlation between the glycoalbumin/glycohemoglobin ratio (GA/HbA1c) and common logarithms of the percentage of total WMH divided by intracranial volume (WMH/IC).

We found no significant association between HbA1c and WMH/IC (*p* = 0.139 for total WMH/IC and *p* = 0.176 for PVH/IC) and between GA and WMH/IC (*p* = 0.144 for total WMH/IC and *p* = 0.099 for PVH/IC). However, GA/HbA1c was significantly and positively associated with both the total WMH/IC and PVH/IC (*p* < 0.001 for both; **Figure 3**) but not with DWMH/IC (data not shown).

### Association between WMH and BMI

**FIGURE 4:**
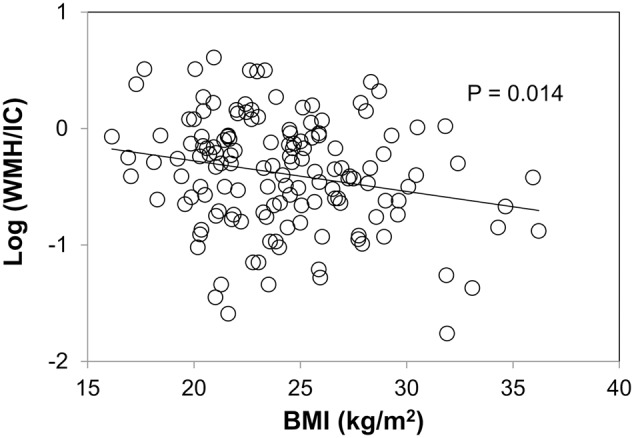
Correlation between body mass index (BMI) and common logarithms of the percentage of total WMH divided by intracranial volume (WMH/IC).

Body mass index was negatively associated with both the total WMH/IC and PVH/IC (*p* = 0.014 for total WMH/IC and *p* = 0.013 for PVH/IC; **Figure 4**) but not with DWMH/IC (data not shown).

### Association between WMH and Blood Pressure

**FIGURE 5:**
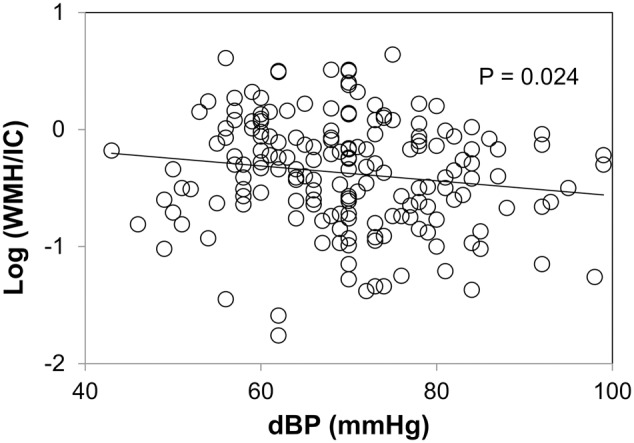
Correlation between diastolic pressure and common logarithms of the percentage of total WMH divided by intracranial volume (WMH/IC).

Plots between WMH and diastolic blood pressure are shown in **Figure 5**. We did not find any correlations between WMH/IC and systolic or pulse pressure (data not shown). Of note, however, diastolic pressure was significantly and negatively correlated with both the total WMH/IC and PVH/IC (*p* = 0.024 for total WMH/IC and *p* = 0.017 for PVH/IC). No significant correlations were found between DWMH/IC and blood pressure (data not shown).

### Multiple Regression Analysis for Identifying the Determining Factor for WMH

The results of the multiple regression analysis are shown in **Table 2**. WMH/IC was significantly and negatively correlated with GA/HbA1c, even after adjustment for age, sex, BMI, and diastolic pressure (*p* = 0.025 for total WMH/IC and *p* = 0.019 for PVH/IC).

**Table 2:** Multiple regression analysis of factors related to total white matter hyperintensity volumes divided by intracranial volumes (WMH/IC).

Covariates	β	*p*-value
Age (y)	0.133	0.158
Sex (*M* = 1, *F* = 2)	0.020	0.803
BMI (kg/m^2^)	–0.091	0.309
Diastolic BP (mmHg)	–0.074	0.408
GA/HbA1c	0.206	0.025^∗^

*BMI, body mass index; BP, blood pressure; GA/HbA1c, glycoalbumin/glycohemoglobin ratio. ^∗^*p* < 0.05.*

### Association of GA/HbA1c with Cognitive Function and ADL

**FIGURE 6:**
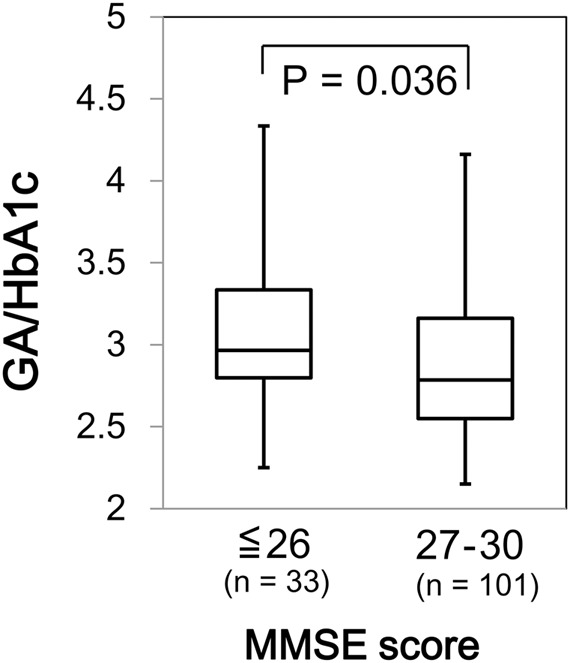
Box plots of the glycoalbumin/glycohemoglobin ratio (GA/HbA1c) compared with Mini–Mental State Examination (MMSE) score categories. Boxes represent the 25th and 75th percentiles, the bands inside the boxes represent medians, and the whiskers represent the full ranges of distributions.

**FIGURE 7:**
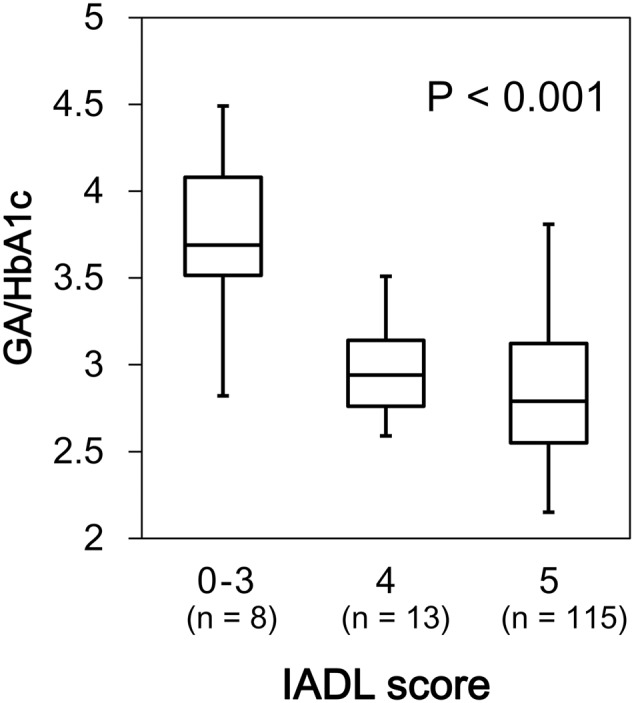
Box plots of the glycoalbumin/glycohemoglobin ratio (GA/HbA1c) compared with IADL score categories. Boxes represent the 25th and 75th percentiles, the bands inside the boxes represent medians, and the whiskers represent the full ranges of distributions.

We found a significant difference in GA/HbA1c among the MMSE categories (*p* = 0.036; **Figure 6**). However, HbA1c (*p* = 0.944) and GA (*p* = 0.085) were not associated with MMSE (data not shown). Similarly, GA/HbA1c was significantly and negatively associated with the IADL categories (*p* < 0.001; **Figure 7**). Additionally, there was a significant difference in GA (*p* = 0.038), but no difference was found in HbA1c (*p* = 0.441, data not shown).

### Binomial Logistic Regression Analyses for Identifying the Determining Factor for MMSE and IADL

In the analysis with MMSE categories as the outcome variable, age (*p* = 0.012) and male sex (*p* = 0.023) were identified as significant risk factors for low MMSE values, whereas neither the common logarithm of WMH/IC nor GA/HbA1c was a significant determining factor. On the other hand, in the analysis with IADL categories as the outcome variable, GA/HbA1c (*p* = 0.006) was identified as the most significant risk factor for low IADL, and its significance was greater than that of age (*p* = 0.056) and the common logarithm of WMH/IC (*p* = 0.051).

## Discussion

In this cross-sectional study, we found significant negative associations between WMH volume and MMSE and between WMH and IADL. Additionally, we found significant correlations between WMH volume and GA/HbA1c (a surrogate marker for glucose variability), BMI, and diastolic pressure. Among the factors studied, GA/HbA1c was a stronger determinant for WMH volume than BMI or diastolic pressure. Furthermore, we found that GA/HbA1c was negatively associated with both MMSE and IADL.

In this study, we used SNIPER to evaluate the WMH volumes. This system can measure WMH quantitatively, with continuous values, and has advantages over the conventional visual rating scales in terms of speed, accuracy between measurements, and sensitivity. The median values of log_10_ (WMH/IC) corresponding to 1, 2, and 3 on the Fazekas scale are approximately -0.5, 0.1, and 0.5, respectively, calculated from the values in our previous report (Ogama et al., 2017).

Although Debette and Markus (2010) reported in a systematic review that WMH has a positive association with the incidence of dementia in the general population, there has been no clear evidence that WMH is associated with cognitive function in elderly patients with DM, and to our knowledge, this is the first report to show a significant association in patients with DM. We found a relatively weak correlation between WMH volume and MMSE possibly because of the small sample size and the subjects’ backgrounds in our study. Our participants showed highly preserved intellectual ability, with a median MMSE score of 29, and there were only few patients with a MMSE score indicative of dementia. Additionally, MMSE might have been an inappropriate marker for distinguishing normal individuals and those with mild cognitive impairment (MCI). For the subjects of our study (most subjects had cognitive functions that were fairly preserved), other tests, such as the Montreal Cognitive Assessment (MoCA), might have been more preferable to identify patients with MCI and might have shown a significant correlation with WMH.

White matter hyperintensity and IADL were significantly correlated, despite our small sample size and the fact that the majority of our patients scored full marks in the IADL test (5 points). From these results, we speculate that WMH could influence IADL impairment in the very early phase. Although we could not convert the 1-point loss in our IADL scale to indices of frailty, such as the Clinical Frailty Scale (Rockwood et al., 2005), it is suspected that patients with “4 points” in our IADL assessment could reflect the state of frailty and WMH could be an image marker for frailty.

Recently, it has been shown that large glucose variability could induce oxidative stress and could be a risk factor for the progression of diabetic complications. Considering that increased glucose variability is negatively correlated with endothelial function (Torimoto et al., 2013) and that some studies have shown the influence of glucose variability on atherosclerosis in type 2 DM patients (Mo et al., 2013), it is possible that glucose variability could also induce WMH progression. We used GA/HbA1c as a marker of glucose variability. Because of the far shorter turnover cycle of albumin than hemoglobin in the blood, GA has been shown to be a more sensitive marker for detecting glucose fluctuation and postprandial glucose excursions than HbA1c (Koga, 2014). Furthermore, GA/HbA1c, which is calculated by dividing GA by HbA1c, can more accurately reflect glucose variability than GA itself (Ogawa et al., 2012). Using the GA/HbA1c index, we found a positive association between WMH and GA/HbA1c but not HbA1c. This result indicates that not the average glucose control level but glucose variability is a more important determining factor for the severity of WMH. Indeed, some studies on type 2 DM showed that HbA1c levels were not associated with WMH volumes (Manschot et al., 2006; de Bresser et al., 2010), and these findings were compatible with our results. To our knowledge, this is the first report to indicate that glucose variability is associated with WMH volumes.

Interestingly, we found a negative correlation between WMH volume and BMI, which is contradictory to the results of previous reports (Xu et al., 2013; Pasha et al., 2017). One of these reports used diffusion tensor imaging to show that a high BMI is related to loss of white matter integrity (Xu et al., 2013). The reason for this difference could be attributed to the participants’ backgrounds. The subjects in the previous studies were considerably younger than the subjects in our study. Moreover, some of the subjects in our study had a BMI < 20 kg/m^2^, which is considered to indicate malnourishment. Recently, the harmful effects of low BMI in the elderly have been reported. For example, Qizilbash et al. (2015) showed that underweight is a risk factor for cognitive decline in the elderly. It is speculated that malnutrition could also exacerbate microvessel vulnerability, and our results could indicate that malnutrition is a risk factor for WMH in elderly patients with DM, rather than of obesity. Indeed, a recent report has shown that the severity of WMH is significantly correlated with nutritional status evaluated using Mini Nutritional Assessment (MNA) in the elderly (de van der Schueren et al., 2016), which could support our results.

We found an inverse correlation between diastolic pressure and WMH volume. High blood pressure has been identified as a major risk factor for the progression of WMH. As for diastolic blood pressure, Gutierrez et al. (2015) showed in the Northern Manhattan Study that WMH volumes are positively associated with high diastolic blood pressure, and this result is completely the opposite of our result. However, impaired cerebrovascular hemodynamic is recognized as a key factor in the development of microvascular diseases (Purkayastha et al., 2014), which could account for the discrepancy in the results between the study by Gutierrez et al. (2015) and our study. Specifically, the baseline age and prevalence of DM were lower in the Northern Manhattan Study than in our study (68 years vs. 77 years and 18% vs. 100%, respectively). In many elderly and diabetic patients (likely due to the progression of atherosclerotic changes and diabetic neuropathy in the latter case), cerebral auto-regulatory capacity is impaired (Kim et al., 2008) and the vascular reserve volume may be low, especially in conditions with low diastolic pressure. Moreover, the rate of our patients treated with antihypertensive drugs was high (65%). Kim et al. (2011) reported that treatment of patients with diabetic vascular complications using antihypertensive drugs induced a continuous decrease in the cerebral blood flow velocity. From this point of view, our results suggest that excessive antihypertensive treatment should not be recommended for preventing cerebral microvascular diseases in elderly patients with DM. Indeed, a recent report showed that in very old hypertensive patients(≥80-years-old), the increase in WMH volume was large in those who had received excessive blood pressure-lowering treatment (Peng et al., 2014).

Although we found positive correlations between WMH and both MMSE and IADL, and between GA/HbA1c and WMH, glucose variability itself could be a risk factor for cognitive dysfunction and IADL decline. Indeed, in a cross-sectional study, it was shown that glucose variability was negatively correlated with MMSE scores (Zhong et al., 2012). In this study, we found significant positive associations of GA/HbA1c with cognitive dysfunction and IADL decline. The former result is compatible with the result of a previous study by Kinoshita et al. (2016), which showed that GA/HbA1c is associated with MMSE and the Revised Hasegawa Dementia Scale. However, to our knowledge, this is the first report showing an association between glucose variability and functional decline in elderly patients with DM.

In binomial logistic regression analyses, we could not find greater significant effects of WMH and GA/HbA1c on low MMSE compared with the effects of age and male sex; however, this negative result might be attributed to the ambiguity of MMSE scoring as described above. On the other hand, it is worthy to note that GA/HbA1c had a significant association with low IADL even after adjustments with age, sex, and WMH. This result indicates that GA/HbA1c is a more influential factor than WMH. However, although there was no statistical significance, the *p*-value of WMH was small, and it is possible that glucose variability and WMH partly have direct effects on low ADL, independent of each other. A further longitudinal study is needed to clarify the roles of WMH and glucose variability on the progression of MCI, dementia, frailty, and ADL dysfunction in these populations.

The present study has several limitations. First, because this was a cross-sectional study, the long-term influence of WMH on cognitive decline and ADL dysfunction were unknown. The results of a longitudinal study are highly awaited. Additionally, the causal relationship between WMH and BMI, glucose variability, or diastolic blood pressure has not been fully elucidated. Whether these factors could actually affect the progression of WMH should be clarified in further longitudinal observational studies. Second, the meaning of the categorizations of PVH and DWMH is unclear. In this study, we observed associations of WMH with IADL, GA/HbA1c, BMI, and diastolic pressure in total WMH and PVH but not in DWMH. However, the automatic segmentation program of SNIPER recognized the majority of WMH volumes as PVH, and the relative volumes of DWMH were negligible. Moreover, DeCarli et al. (2005) suggested that total WMH, PVH, and DWMH are highly correlated with each other and that the classifications of PVH and DWMH are arbitrary. Thus, the meaning of the categorizations of PVH and DWMH might be of little importance. Third, we performed only simple tests for evaluating cognitive function, nutritional status, and glucose variability, i.e., MMSE, BMI, and GA/HbA1c, respectively. As described above, few subjects had obvious cognitive impairment in this population, whereas it is estimated that a substantial number of patients with MCI were included. MMSE might be inappropriate for identifying MCI, and MoCA might have been more preferable. Additionally, we should have performed detailed measurements, such as MNA, continuous glucose monitoring, and home/ambulatory blood pressure measurements, to confirm our results with regard to nutritional status, glucose variability, and blood pressure decrease, respectively.

## Conclusion

We found a significant negative association between WMH and cognitive function and between WMH and IADL status in a group of elderly patients with DM. Furthermore, we found a significant association of WMH with GA/HbA1c, BMI, and diastolic blood pressure. Glucose variability is the most influential factor, and the avoidance of glucose fluctuations may be a key strategy to prevent WMH and the further progression of cognitive impairment and IADL decline in elderly patients with DM.

## Ethics Statement

This study was carried out in accordance with the recommendations of Ethical Guidelines for Medical and Health Research Involving Human Subjects, Ministry of Education, Culture, Sports, Science and Technology and Ministry of Health, Labour and Welfare, Japan, with written informed consent from all subjects. All subjects gave written informed consent in accordance with the Declaration of Helsinki. The protocol was approved by the ethical committee of Tokyo Metropolitan Geriatric Hospital.

## Author Contributions

YoT, TS, and AA designed the study, analyzed data, and wrote the draft of the manuscript. YK, TY, KS, YuT, RK, YC, and SM contributed to data collection, analysis and interpretation of data. YF, AT, and HI contributed to data interpretation, and critically reviewed the manuscript.

## Conflict of Interest Statement

The authors declare that the research was conducted in the absence of any commercial or financial relationships that could be construed as a potential conflict of interest.
